# Ethylene and epoxyethane metabolism in methanotrophic bacteria: comparative genomics and physiological studies using Methylohalobius crimeensis

**DOI:** 10.1099/mgen.0.001306

**Published:** 2024-10-25

**Authors:** Noah Toppings, Meghan Marshall, Angela V. Smirnova, Andriy Sheremet, Anthony S. Pasala, Felix C. Nwosu, Morgan Hepburn, Ian Lewis, Nicholas V. Coleman, Peter F. Dunfield

**Affiliations:** 1 Department of Biological Sciences, University of Calgary, Calgary, Alberta, Canada; 2 School of Natural Sciences, Macquarie University, New South Wales, Australia; 3 ARC Centre of Excellence in Synthetic Biology, Macquarie University, New South Wales, Australia

**Keywords:** alkene, epoxide, epoxyethane, ethene, ethylene, methane, methanotroph, Coenzyme M

## Abstract

The genome of the methanotrophic bacterium *Methylohalobius crimeensis* strain 10Ki contains a gene cluster that encodes a putative coenzyme-M (CoM)-dependent pathway for oxidation of epoxyethane, based on homology to genes in bacteria that grow on ethylene and propylene as sole substrates. An alkene monooxygenase was not detected in the *M. crimeensis* genome, so epoxyethane is likely produced from co-oxidation of ethylene by the methane monooxygenase enzyme. Similar gene clusters were detected in about 10% of available genomes from aerobic methanotrophic bacteria, primarily strains grown from rice paddies and other wetlands. The sparse occurrence of the gene cluster across distant phylogenetic groups suggests that multiple lateral gene transfer events have occurred in methanotrophs. In support of this, the gene cluster in *M. crimeensis* was detected within a large genomic island predicted using multiple methods. Growth studies, reverse transcription-quantitative PCR (RT-qPCR) and proteomics were performed to examine the expression of these genes in *M. crimeensis*. Growth and methane oxidation activity were completely inhibited by the addition of >0.5% (v/v) ethylene to the headspace of cultures, but at 0.125% and below, the inhibition was only partial, and ethylene was gradually oxidized. The *etnE* gene encoding epoxyalkane:CoM transferase was strongly upregulated in ethylene-exposed cells based on RT-qPCR. Proteomics analysis confirmed that EtnE and nine other proteins encoded in the same gene cluster became much more predominant after cells were exposed to ethylene. The results suggest that ethylene is strongly inhibitory to *M. crimeensis*, but the bacterium responds to ethylene exposure by expressing an epoxide oxidation system similar to that used by bacteria that grow on alkenes. In the obligate methanotroph * M. crimeensis*, this system does not facilitate growth on ethylene but likely alleviates toxicity of epoxyethane formed through ethylene co-oxidation by particulate methane monooxygenase. The presence of predicted epoxide detoxification systems in several other wetland methanotrophs suggests that co-oxidation of ambient ethylene presents a stress for methanotrophic bacteria in these environments and that epoxyethane removal has adaptive value.

**Impact Statement**

Methane is a major greenhouse gas and the primary target of short-term efforts to combat climate change. Methanotrophic bacteria effectively limit potential methane emissions from many natural and anthropogenic environments, and thus, it is valuable to fully understand the physiological diversity and environmental tolerances of these bacteria. Here, we present evidence that an aerobic methanotrophic bacterium (*Methylohalobius crimeensis*) may oxidize epoxyethane, a toxic byproduct of the unavoidable co-oxidation of ethylene by methane monooxygenase enzymes. The proposed pathway used to remove epoxyethane is similar to that used by bacteria able to grow on alkenes as sole carbon and energy sources, but in *M. crimeensis*, this pathway appears to function primarily in detoxification. The genes encoding this pathway are present in about 10% of sequenced methanotroph genomes, suggesting that it has an adaptive value in certain habitats. These results expand our view of the physiological capabilities of methanotrophs and their adaptation to different environments. The findings could be relevant, for example, in the design of methane biofiltration systems.

## Data Summary

The genome of *Methylohalobius crimeensis* is available as JGI-IMG Taxon ID 2524614657 (Joint Genome Institute Integrated Microbial Genomes) and NCBI Taxon ID 1283300 (National Center for Biotechnology Information). Other public domain genomes from these databases were used as indicated in the ‘Results’ section. The proteomics data generated in this study have been submitted to the EMBL-EBI Pride database (accession number PXD05161).

## Introduction

Most aerobic methanotrophs are extreme specialists, capable of utilizing only methane and its degradation products as their energy sources [1,2]. However, a few species of ‘facultative methanotrophs’, particularly species of *Methylocella*, *Methylocystis*, *Methylocapsa* and *Methylacidiphilum*, are able to metabolize some other simple substrates for energy, including some alkanes, alcohols, organic acids, aldehydes and ketones up to C3 in length [2,4]. Some methanotrophs can even grow lithotrophically on reduced sulphur compounds, CO or H_2_ [5,7]. The known metabolic diversity of methanotrophs has been steadily growing since the discovery of the first facultative methanotroph in 2005 [1].

The first step in methanotrophy, the conversion of methane into methanol, is catalysed by methane monooxygenase (MMO). MMO enzymes exist in two known forms, a particulate membrane-bound form (pMMO) and a soluble cytoplasmic form (sMMO). Both display a certain amount of promiscuity in their substrate selection and can co-oxidize a variety of short-chain alkanes, halogenated alkanes, alkenes and aromatic compounds [8]. Methanotrophs using sMMO generally co-oxidize a wider range of compounds, and at a faster rate, than those using pMMO, suggesting that the sMMO is the more promiscuous enzyme [8]. In general, non-methane compounds are oxidized to incidental byproducts that do not contribute to cellular energy production, although the co-oxidation of propane by MMOs in the facultative methanotrophs *Methylocella silvestris* and *Methylacidiphilum caldifontis* does result in catabolic energy gain through downstream pathways of propanol oxidation [3,9].

The fundamental promiscuity and adaptability of the MMO enzymes is also evident when looking at the diverse evolutionary lineages to which each enzyme belongs. sMMO belongs to the soluble di-iron-containing multicomponent monooxygenase family (SDIMO), which includes enzymes adapted to act on aromatic hydrocarbons, short-chain alkenes and short-chain alkanes in addition to methane [10]. Similarly, the copper membrane monooxygenase (CuMMO) enzyme family includes pMMO, ammonia monooxygenase and a few short-chain alkane monooxygenases [11]. Some CuMMOs have also been reported to be adapted as ketone monooxygenases [3] or alkene monooxgyenases [12]. Most CuMMOs can co-oxidize several structurally similar substrates but usually show clear specialization to one particular substrate [8,13]. For example, pMMO will oxidize ammonia and some alkanes, but with a lower affinity and lower reaction rates than for methane. Both CuMMOs and SDIMOs have, therefore, evolved to specialize on particular substrates, and distinct phylogenetic clusters of the enzymes that prefer particular substrates can be delineated [10,11].

Ethylene is a trace atmospheric gas that is co-oxidized to epoxyethane by MMO enzymes [14,17]. Ethylene is produced in the environment abiotically through the combustion of fossil fuels and organic wastes [18] and biotically by diverse plants, bacteria, fungi and algae [19,21]. It is present in atmospheric mixing ratios that vary locally from about 150 ppbv [22] to 1 ppmv [23], but levels can be much higher in soil pore gas, ranging from 1 [24] to 15 ppmv [25]. In some non-methanotrophic soil bacteria, ethylene oxidation to epoxyethane (or ethylene oxide) is the initial step in a catabolic pathway that can support growth [26,28]. However, despite the MMO-catalysed epoxidation step, no methanotroph has been shown to grow on ethylene. In fact, ethylene is often toxic to methanotrophs, likely due to the damaging effects of the highly reactive epoxyethane [29,30]. Ethylene mixing ratios of only 3–50 ppmv strongly inhibited methane oxidation activity in several forest soils [31,32], and gene and transcript counts of *pmoA* (encoding a subunit of the pMMO) in a rice paddy soil were reduced by high dosages of ethephon, a precursor of ethylene [33].

**Fig. 1.**
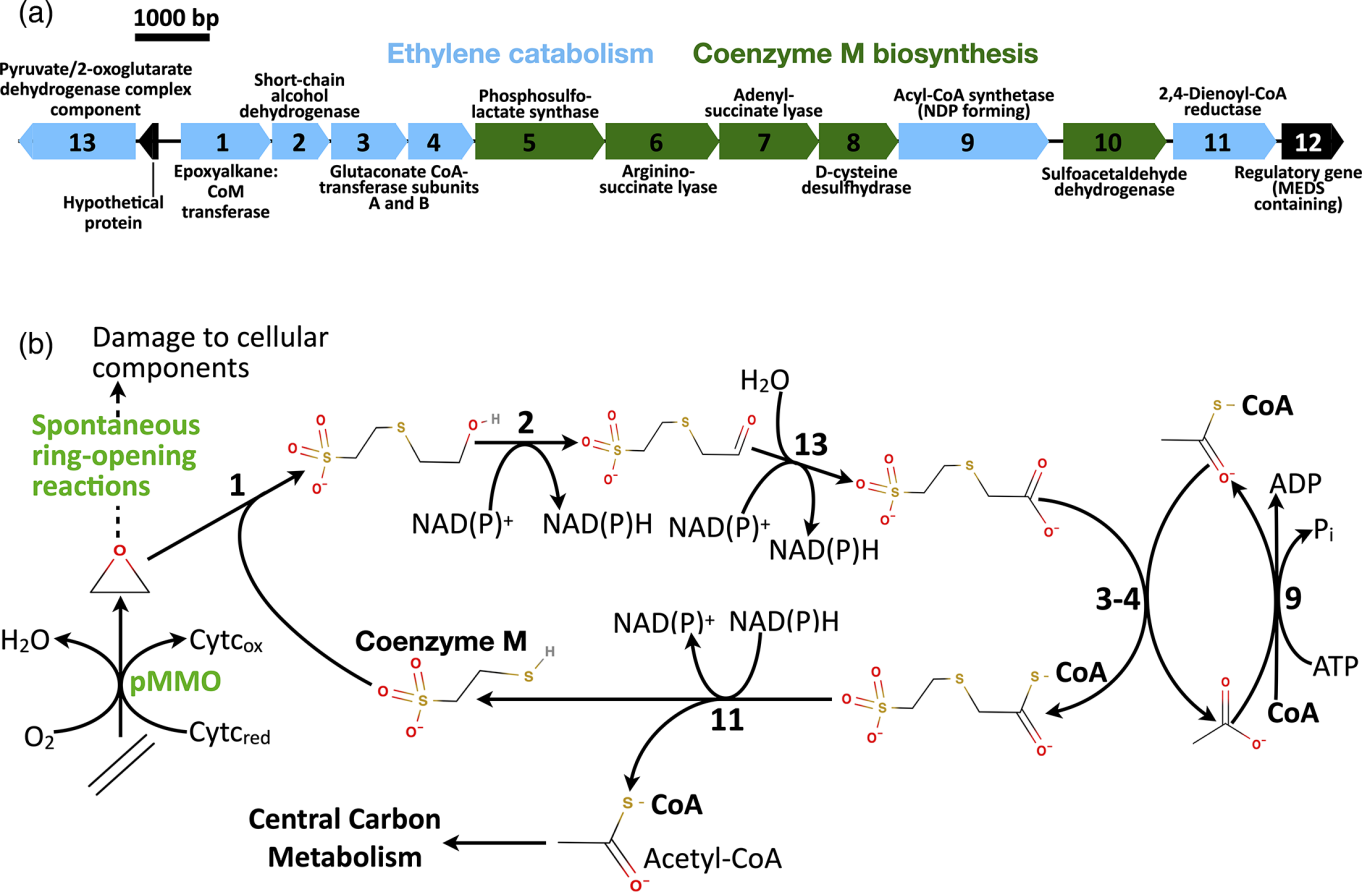
Predicted genes and pathway of ethylene metabolism in *M. crimeensis* 10Ki^T^. (a) Organization of the gene cluster predicted to encode coenzyme-M (CoM) biosynthesis and epoxide metabolism. (b) Predicted pathway of ethylene metabolism, adapted from Ensign and Allen [27], Coleman and Spain [26] and Mattes *et al.* [28].

*Methylohalobius crimeensis* is the most halophilic methanotroph known [34]. The type strain of *M. crimeensis*, strain 10Ki, was originally isolated from a hypersaline lake in the Crimea Peninsula of Ukraine [34]. *M. crimeensis* are mesophiles (optimum 30 °C), neutrophiles (optimum 6.5–7.5 pH) and moderate halophiles (optimum 1–1.5 M NaCl). The genome (JGI-IMG Taxon ID 2524614657; NCBI Taxon ID 1283300) encodes a typical methanotrophic enzyme repertoire, including a pMMO enzyme for the initial step in methane oxidation [35]. There is little genomic evidence for energy conservation from other substrates, and these bacteria are, therefore, thought to be typical obligate methanotrophs that grow on methane and methanol only. However, we noticed that the *M. crimeensis* genome contains a gene cluster predicted to encode a pathway for the oxidation of epoxyethane to acetyl-CoA (Fig. 1). Here, we describe our investigation of ethylene oxidation in *M. crimeensis*, along with comparative genomics studies with other methanotrophs.

## Methods

### Growth experiments

Liquid medium S was prepared according to Heyer *et al.* [36], except that LaCl_3_•7H_2_O was added at the same molar concentration as copper. For growth of starter cultures, 1 L serum bottles containing 300–400 ml of sterilized medium were inoculated with * M. crimeensis* 10Ki^T^ from frozen stock and capped with butyl rubber septa. CO_2_ (5% v/v) and CH_4_ (20% v/v) were added via syringe through sterile 0.22 µm filters, and cultures were incubated at 28 °C while rotary shaking at 110 r.p.m.

To initially determine ethylene tolerance, starter cultures were grown on methane to an OD_600_ of about 0.15, and then, ethylene was added at headspace mixing ratios of 0.1–5% (v/v), with and without methane. Growth was monitored by measuring the OD at 600 nm (OD_600_), and hydrocarbon oxidation activities were monitored by gas chromatography (see below). To assess the oxidation of ethylene at lower mixing ratios, cultures were grown to OD_600_ of 0.15 on methane and then uncapped for 15 min to aerate the headspaces. The headspaces of the bottles were then reconstituted with the following treatments (in triplicate): (i) 8–10% v/v CH_4_+0.125% v/v C_2_H_4_, (ii) 8–10% v/v CH_4_ only and (iii) 0.125% v/v C_2_H_4_ only. All bottles contained a balance of air+5.0% v/v CO_2_. In addition, three replicates were autoclaved as sterile controls (+CH_4_ +C_2_H_4_), and three bottles containing uninoculated medium were included as blanks (+CH_4_ +C_2_H_4_). Bottles showing depletion of ethylene were replenished with the appropriate volumes of methane and ethylene once after these were depleted. To test whether ethylene had any effect on net growth, this experiment was repeated using 100 ml cultures in 250 ml serum bottles, with only a single addition of ethylene. Samples were taken at intervals to determine OD_600_, DNA and protein concentrations and headspace gas mixing ratios as described below.

### Gas chromatography

Headspace mixing ratios of CH_4_, C_2_H_4_ and CO_2_ were measured via GC on a Model 8610C gas chromatograph (SRI Instruments, Torrance, CA, USA) equipped with a flame ionization detector (2 m HayeSep-D column, column 190 °C, detector 300 °C and N_2_ as carrier gas) and a methanizer. To ensure that incubations remained aerobic, O_2_ was estimated with an SRI/Valco 140BN ^63^Ni electron capture detector on the same GC system. The volume of headspace injected into a 100 µl sample loop was 2.0 ml. The sample loop was flushed with 33 ml of air between each sample/standard. Peaks were standardized using a commercially certified standard containing 0.5 % CH_4_ and 1.0 % CO_2_ in N_2_ (Linde, Mississauga, ON, Canada) or a 0.10% C_2_H_4_ standard made by adding 0.10 ml of >99.5% pure C_2_H_4_ (Linde) into a 1 l flask capped with a butyl stopper.

### Growth measurements

OD_600_ values were measured on 1 ml samples in disposable plastic cuvettes using an Amersham Biosciences (Bath, UK) Ultrospec 10 Cell Density Meter. To measure biomass (dry weight), 20 ml samples were centrifuged in a Beckman Coulter (Brea, CA, USA) Avanti J-E Centrifuge for 15 min at 8000 ***g***. The supernatant was discarded, and the samples were dried overnight at 68 °C. Samples were allowed to cool for 30 min at room temperature before being weighed on an analytical balance. To quantify DNA and protein, 10 ml samples were centrifuged as above and then frozen at −80 °C until processing. For DNA, cell pellets were thawed and resuspended in 200 µl of PBS, homogenized using a Precellys 24 Bead Mill Homogenizer (Bertin Instruments, Montigny-le-Bretonneux, France) and extracted using the FastDNA Extraction Kit for Soil (MP Biomedicals, Santa Ana, CA, USA). For protein, cell pellets were resuspended in 500 µl of PBS, and the protein was precipitated with 50 µl of cold TCA (100%), then denatured by boiling at 99 °C for 8 min and centrifuged at 15 900 ***g*** for 5 min, and the protein pellet was resuspended in 200 µl of 0.1 M NaOH. An Invitrogen Qubit fluorometer (Thermo Fisher, Waltham, MA) was used to quantify both DNA and protein samples, using settings Quant-iT DNA HS and Quant-iT Protein (Thermo Fisher).

### RT-qPCR

To determine gene expression of *etnE* [encoding 2-hydroxypropyl-coenzyme-M (CoM) lyase] in *M. crimeensis* grown with or without 0.125% ethylene, 45 ml liquid samples were taken from cultures after 0, 4 and 15 days. Samples were immediately placed on ice, and 5 ml of stop solution was added (5% buffer-equilibrated phenol at pH 7.3 in ethanol). Samples were centrifuged at 8000 ***g*** at 4 °C for 15 min, supernatants were discarded, and cell pellets were frozen in liquid nitrogen and stored at −80 °C. RNA was extracted using the RNAqueous 4PCR DNA-free RNA isolation for RT-PCR kit (Thermo Fisher). The concentration of RNA was measured using a Qubit fluorometer and a Quant-iT RNA kit (Thermo Fisher). The RNA samples were then either used immediately or frozen at −80 °C until use in cDNA synthesis.

RNA from two experimental replicates (separate bottles) of each treatment (CH_4_ +C_2_H_4_, CH_4_ only and C_2_H_4_ only) was converted into cDNA using 4 µl of 5×iScript RT Supermix (Bio-Rad, Hercules, CA) and variable volumes of RNA template and nuclease-free distilled water up to a total volume of 16 µl, normalized to the lowest concentration of RNA template in any sample. A negative control was performed using the No-RT Control Supermix on the sample with the highest RNA concentration. The mixtures were run in an Applied Biosystems 2720 Thermal Cycler (Thermo-Fisher) using a program of 25 °C for 5 min, 46 °C for 20 min and 95 °C for 1 min.

Geneious Prime software (Biomatters Inc., Auckland, New Zealand) was used to design two reverse transcription-quantitative PCR (RT-qPCR) primer sets targeting the *etnE* of *M. crimeensis* (Table S1, available in the online Supplementary Material). Gyrase was used as an endogenous reference single-copy housekeeping gene, amplified using *gyrA1* primers [37] that were confirmed to match the target gene in *M. crimeensis* (Table S1). Primer concentrations for all three assays were optimized for *M. crimeensis* and achieved amplification efficiencies of >98.6%. RT-qPCR was performed on the cDNA using 5 µl of SsoAdvanced Universal SYBR Green Supermix (BioRad), 0.25 µl (10 µM) each of forward and reverse primers, 3.5 µl of nuclease-free distilled water and 1 µl of cDNA template per reaction. In addition to the No-RT Supermix negative control, a positive control was performed using *M. crimeensis* genomic DNA as the template, and two other controls were performed with the highest concentration of RNA and nuclease-free water as templates. Each mixture was run in a Qiagen Rotor-Gene Q (Toronto, ON, Canada) with a 3 min initial 95 °C denaturation step, then 35 cycles of denaturing at 95 °C for 10 s and a combined annealing and extension phase of 60 °C for 25 s. Differential gene expression was calculated using the ∆∆Ct method of relative quantification [38], using *gyrA* as the reference.

Statistical analyses were made for each RT-qPCR assay using two-way ANOVAs. Reported significance values are based on a complete model with day and ethylene addition as factors, log-transformed data and multiple comparisons made via the Holm–Sidak method. Multiple configurations of the ANOVA were tested (different transformations, pooling the two +C_2_H_4_ treatments versus treating them as distinct), and despite the limited replication, the effects of ethylene on *etnE* expression were always highly significant (see ‘Results’). Significance was also verified for each comparison using Welch’s unequal variances t-tests.

### Proteomics

Proteomics sample preparation and liquid chromatography–mass spectrometry (LC–MS) data collection were completed at the Calgary Metabolomics Research Facility. Cells exposed to either CH_4_ alone or CH_4_ +C_2_H_4_ for 10 days were harvested and frozen from the same experiment used for RNA extraction (three replicates of each treatment). Frozen cells (−80 °C) were thawed at room temperature and resuspended in a mix of 978 µl of water and 122 µl Tris-SDS buffer (MT buffer, MP Biomedicals, Solon, OH, USA) to lyse cells. Lysed cells were transferred into 2 ml Lysing Matrix E tubes (MP Biomedicals, Solon, OH, USA) and homogenized twice for 40 s at maximum speed in the Precellys 24 homogenizer (ESBE Scientific, Markham, ON, Canada). Cell debris was removed by centrifugation, and the supernatants were retained. Protein concentration in cell lysates was estimated using a NanoDrop Microvolume Spectrophotometer (Thermo Fisher) at 280 nm. Proteins were purified with paramagnetic beads using single-pot, solid-phase-enhanced sample preparation (SP3) technology [39] and digested into peptides overnight at 37 °C using Trypsin/Lys-C Protease Mix (Thermo Fisher). Peptide concentrations in each sample were quantified using a Pierce Colorimetric Quantitative Peptide Assay (Thermo Fisher) and a microplate reader (Molecular Devices, LLC., San Jose, CA, USA). Subsequently, solutions were normalized to 10 µg of peptides in 40 µl and labelled with a specific tandem mass tag (TMT) for each sample with a ratio of 1 : 4. After incubation at room temperature for an hour, peptide solutions were quenched with hydroxylamine, pooled together and purified using an Evolute Express AX column (Biotage, San Jose, CA, USA). Eluted peptides were dried in a vacuum centrifuge overnight, resuspended in 90 µl of 0.1% formic acid, and sonicated for 7 min before being run on an Easy-nLC 1200 coupled to an Orbitrap Fusion Lumos Tribrid instrument with the FAIMS source (Thermo Fisher). Peptides were loaded on an Acclaim PepMap 100 C18 trap column, washed and then separated on a 25 cm × 75 µm EASY-Spray HPLC C18 column with a 90 min 0–35% 80% acetonitrile and 0.1% formic acid (solvent B) gradient. FAIMS CV was set at −40, and MS1 data, 375–1600 m/z, were collected at a resolution of 120K. Data-dependent MS2 scans, with first mass 110 m/z, were collected at a resolution of 50K. MS2 scans were triggered on ions with a charge greater than or equal to 2, with a 0.7 m/z isolation window, and used 38% normalized HCD (higher-energy collisional dissociation) collision energy.

Data were searched with Frag-Pipe [40,41] against the *M. crimeensis* proteome downloaded from the NCBI, annotated by the NCBI Prokaryotic Genome Annotation Pipeline: NCBI RefSeq GCF_000421465.1. The default TMT-10 workflow search parameters were used after the removal of the cysteine carbamidomethylation modification and changing the TMT label type to TMT-11. Protein reporter ion intensity was normalized to the total reporter ion intensity of the TMT channel. Normalized values for proteins were subjected to a differential expression analysis using an open-source R package DESeq2 in the R environment, version 4.3.2 [42]. To visualize differentially expressed proteins, a volcano plot was generated using the ggplot2 package 3.4.4 in the R environment.

### Bioinformatics

Most bioinformatics analyses were performed using the JGI-IMG platform [43]. Potential lateral gene transfer (LGT) was assessed using AlienHunter [44] and IslandPath-DIMOB [45]. The latter is integrated within IslandViewer4 [46]. Mobile genetic elements were detected with mobileOG-db v1.1.3 [47] and refined using JGI IMG annotations. Potential integrative and conjugative elements (ICEs) were screened for using ICEBerg [48]. 16S rRNA gene sequences were extracted from methanotroph genomes in JGI-IMG and NCBI and used to construct a maximum-likelihood phylogeny. Duplicate genes and genes smaller than 800 nucleotides were removed. The multiple sequence alignment was produced with MAFFT v.7.450 [49]. The phylogenetic tree was constructed with FastTree v2.1.11 [50] and visualized using iTOL v5 [51].

## Results

### Genomic evidence for ethylene and epoxyethane metabolism in *M. crimeensis*

Fig. 1 summarizes the predicted oxidation pathway of ethylene and epoxyethane by *M. crimeensis* 10Ki^T^ based on a bacterial pathway described previously [26,28] and the genome comparison to the model alkene-consuming bacteria *Mycobacterium rhodesiae* JS60 (synonym of *Mycolicibacterium rhodesiae* JS60; JGI IMG Taxon ID 2506783048; NCBI Taxon ID 931627) and *Xanthobacter autotrophicus* (Py2 JGI IMG Taxon ID 640753059; NCBI Taxon ID 78245). First, ethylene is hypothesized to be oxidized to epoxyethane (ethylene oxide) by pMMO, encoded by two complete *pmoCAB* operons in the *M. crimeensis* genome (H035DRAFT_0376–0378 and H035DRAFT_2364–2362). No dedicated alkene monooxygenase is encoded in the genome. In fact, only two other monooxygenases are annotated: a fatty acid desaturase/alkane 1-monooxygenase (H035DRAFT_2069) and a lysine monooxygenase (H035DRAFT_2302), both of which likely perform biosynthetic functions. Proteins catalysing the further oxidation of epoxyethane to acetyl-CoA, along with biosynthesis of the dedicated cofactor CoM and a hydrocarbon-responsive regulatory factor, are predicted to be encoded in a single cluster of 13 adjacent genes (Fig. 1, Table S2).

Gene H035DRAFT_2946 is a close homologue of *etnE*, encoding epoxyalkane:CoM transferase (EtnE or EaCoMT). Combined results of iProEP and iPromoter-2L software [52,53] predicted sigma-70 (RpoD)-dependent promoter sequences 112–192 bp and 125–205 bp upstream of the *etnE* gene start (H035DRAFT_2946), with the highest score of 0.995. Neural network prediction [54] indicated the most likely promoter sequence at 120–165 bp upstream. The *etnE* and 11 downstream genes are likely expressed as a single operon. Moreover, Virtual Footprint software predicted putative binding sites for a homologue of the stress response regulator CpxR at positions 116 and 165 bp on the reverse DNA strand [55].

**Fig. 2.**
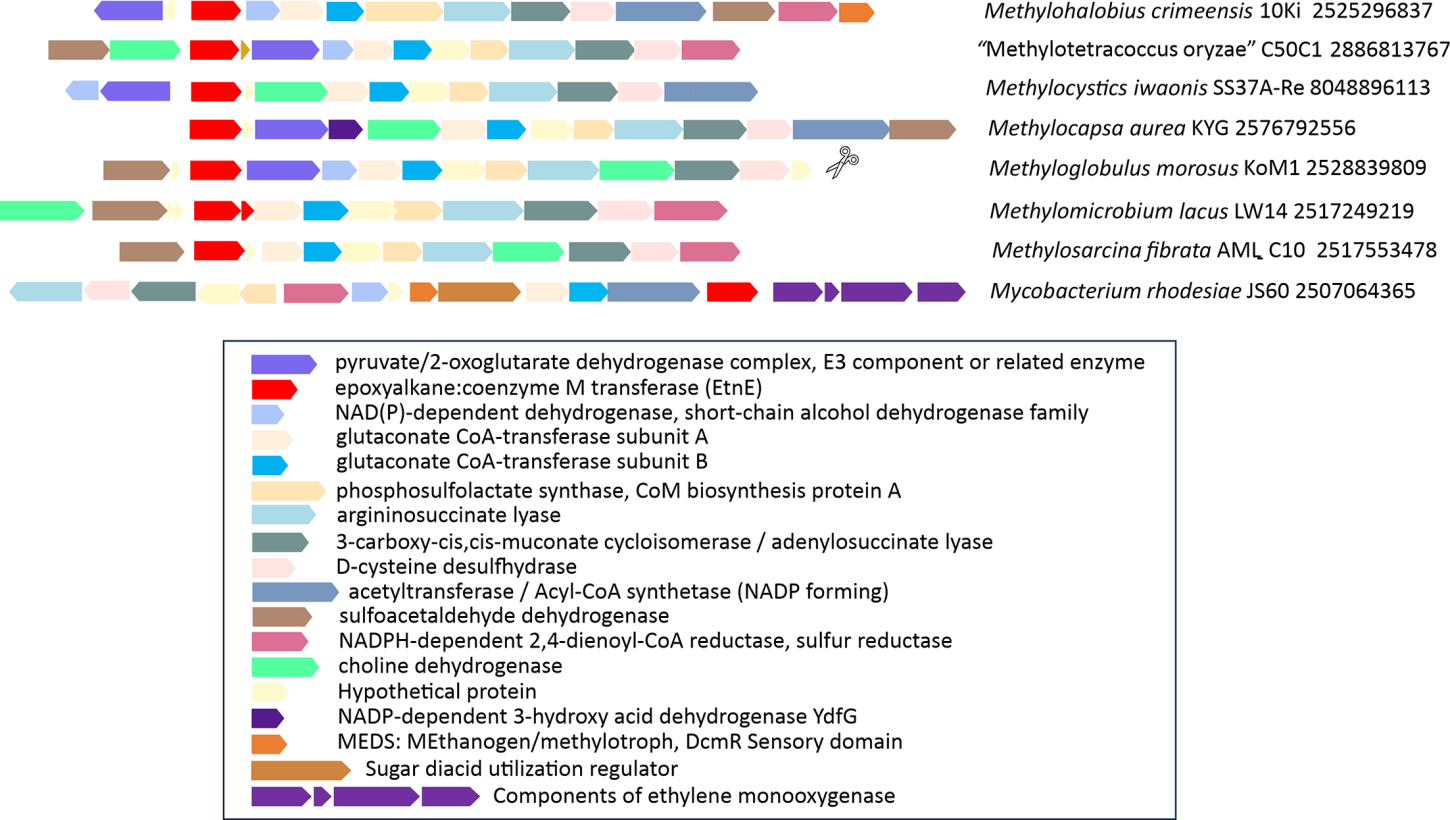
Comparison of putative epoxyethane metabolism/CoM biosynthesis gene clusters in *M. crimeensis*, selected other aerobic methanotrophs and the model ethylene oxidizing actinobacterium *M. rhodesiae* JS60. Genomes and accession numbers for the *etnE* genes are listed to the right. The scissor icon indicates the end of the assembled contig in the genome of strain KoM1. Homologues of the sulfoacetaldehyde dehydrogenase and pyruvate/2-oxoglutarate dehydrogenase E3 components are not contiguous in the *M. rhodesiae* genome but are present nearby, <10 kb distant (Table S2).

The EtnE enzyme requires CoM and performs the first step in epoxide catabolism in model ethylene- and propylene-oxidizing bacteria (Fig. 1). The predicted amino acid sequence of EtnE in *M. crimeensis* shows 47% identity to the EtnE of the well-characterized ethylene-oxidizing bacterium, *M. rhodesiae* JS60 and 77% to two EtnE proteins in the alkene oxidizer *X. autotrophicus* Py2 (Table S2). The *etnE* in *M. crimeensis* is adjacent to genes encoding NAD(P)-dependent short-chain alcohol dehydrogenase, two subunits of a glutaconate CoA-transferase, an acyl-CoA synthetase (nucleoside diphosphate forming), an NADPH-dependent 2,4-dienoyl-CoA reductase and a pyruvate/2-oxoglutarate dehydrogenase complex (on the opposite strand), which together are predicted to encode further reactions that complete the oxidation of epoxyethane to acetyl-CoA as summarized in Fig. 1. These six predicted proteins in *M. crimeensis* show derived amino acid identities of 36–54% to homologues in *Mycobacterium rhodesiae* JS60 (Table S2) and 62–77% to best homologues in *X. autotrophicus* Py2. Although the protein identities to homologues in the proteobacterium *X. autotrophicus* Py2 are higher than to the actinobacterium *M. rhodesiae* JS60, Py2 has multiple copies of most genes, arranged into at least three different clusters in the genome (Table S2). In contrast, the arrangement of all genes into a single cluster in *M. rhodesiae* JS60 is similar to the arrangement in *M. crimeensis* and is shown for comparison in Fig. 2.

Further evidence that *M. crimeensis* is capable of epoxide catabolism is provided by the presence of genes encoding five enzymes required for the biosynthesis of CoM within the same cluster [56]: phosphosulfolactate synthase, argininosuccinate lyase, adenylosuccinate lyase, d-cysteine desulfhydrase and sulfoacetaldehyde dehydrogenase (Fig. 1, Table S2). The encoded proteins show 45–55% predicted amino acid identities to the homologous proteins in *X. autotrophicus* Py2 whose role in CoM biosynthesis has been investigated *in vitro* [56,57]. Only the sulfoacetaldehyde dehydrogenase from *M. crimeensis* has no significant amino acid sequence similarity to its functional analogue in *X. autotrophicus* Py2 (XcbA).

The last protein encoded in the *M. crimeensis* 10Ki^T^ gene cluster shown in Fig. 1 (H035DRAFT_2935) is a regulatory protein containing a MEDS (MEthanogen/methylotroph, DcmR Sensory) domain, along with a helix-turn-helix DNA-binding domain. The MEDS domain is typical of regulatory proteins that bind to simple hydrocarbon derivatives and then transduce regulatory signals [58,59]. The MEDS domain encoded by *M. crimeensis* is homologous to the same domain in a traditional two-component regulatory system that regulates the expression of a similar gene cluster in the ethylene-oxidizing bacterium *M. rhodesiae* JS60 [60].

The epoxyethane oxidation/CoM biosynthesis-encoding genes in *M. rhodesiae* JS60 (and in one of the gene clusters in *X. autotrophicus* Py2) are also adjacent to a set of ethylene/propylene monooxygenase-encoding genes, which are not found in *M. crimeensis* (Fig. 2). In fact, when examining the overall gene cluster encoding epoxide oxidation, CoM synthesis and a regulator, the major difference between *M. rhodesiae* JS60 and *M. crimeensis* 10Ki^T^ is the lack of genes encoding an ethylene monooxygenase in the latter (Fig. 2, Table S2).

### Ethylene tolerance and ethylene oxidation in *M. crimeensis*

**Fig. 3.**
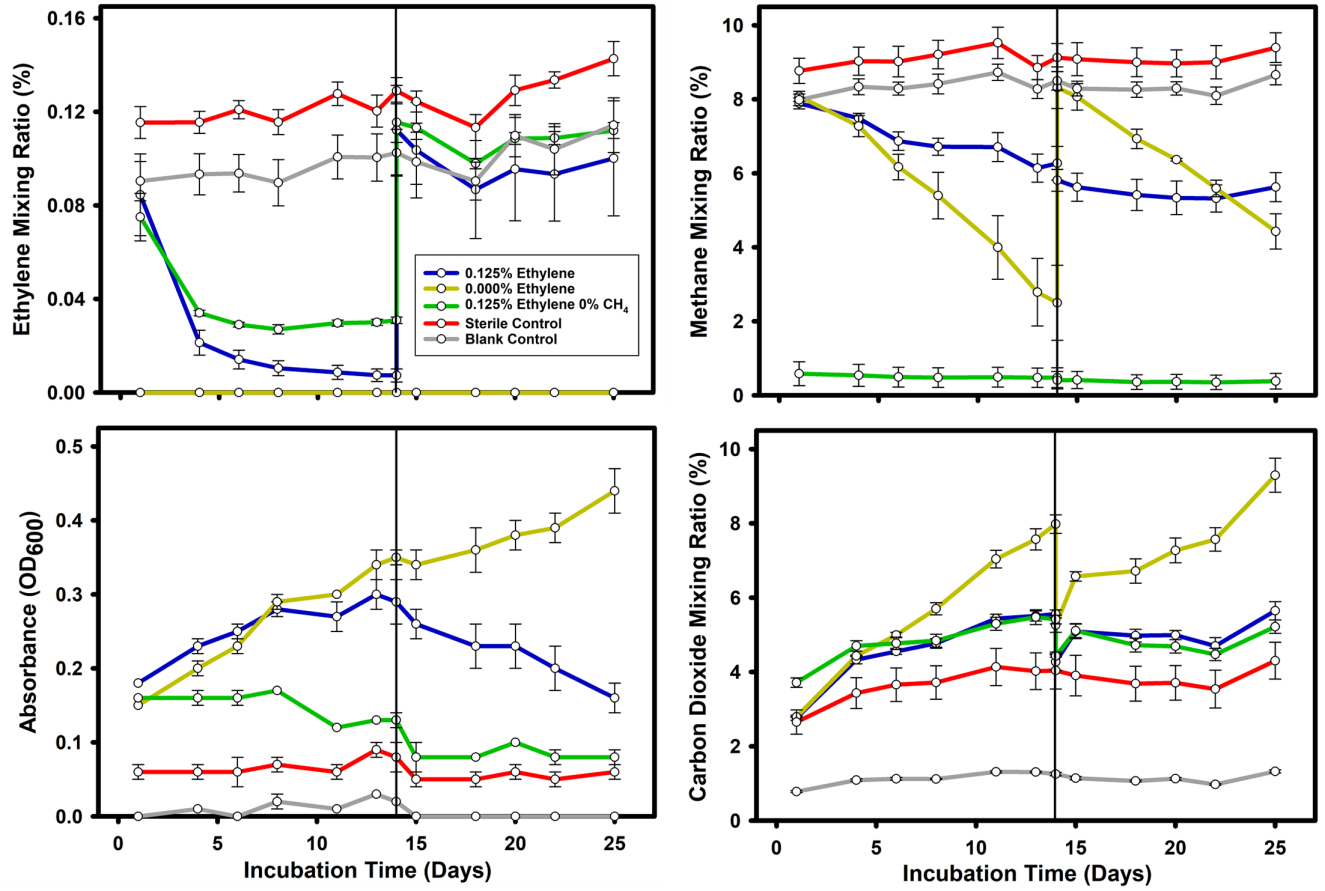
Time courses of methane and ethylene consumption, CO_2_ production and growth of *M. crimeensis* exposed or not exposed to 0.125% ethylene. Cells were pre-grown with methane to an initial OD_600_ of 0.15 (day 0) and then exposed to 0% or 0.125% C_2_H_4_ on day 0 and again on day 14 (vertical black lines). Data points are the mean of three biological replicates ±1 sem.

Adding ethylene at 0.5–5% (v/v) to mid-log phase cultures growing on methane resulted in cessation of growth (Fig. S1A) and complete loss of methane oxidation activity (Fig. S1B). However, when smaller amounts of C_2_H_4_ (0.035% or 0.125%) were added, methane oxidation activity continued (at least temporarily) and C_2_H_4_ was oxidized. A representative experiment with 0.125% C_2_H_4_ is shown in Fig. 3. The added C_2_H_4_ was gradually consumed in treatments with or without CH_4_, but C_2_H_4_ consumption was more rapid when CH_4_ was also present. In cultures initially grown on methane, growth (based on OD_600_) immediately ceased upon transfer to a headspace containing ethylene without methane. However, growth continued in the cultures provided with both CH_4_ and C_2_H_4_, albeit at slower rates compared with cultures growing optimally on CH_4_ alone. A second addition of C_2_H_4_ resulted in a complete loss of methane oxidation activity and a gradual decline in OD_600_ (Fig. 3). These results show that the bacteria could not grow on C_2_H_4_ alone. Instead, C_2_H_4_ inhibited both cell growth and CH_4_ oxidation. This inhibition could be partially relieved in the presence of CH_4_, which is needed to drive growth and generate the energy needed for the detoxification of epoxyethane. The better outcomes in the presence of CH_4_ are also consistent with competitive inhibition, where the CH_4_ limits access of C_2_H_4_ to the pMMO active site and, therefore, should also limit the production of toxic epoxyethane.

**Fig. 4.**
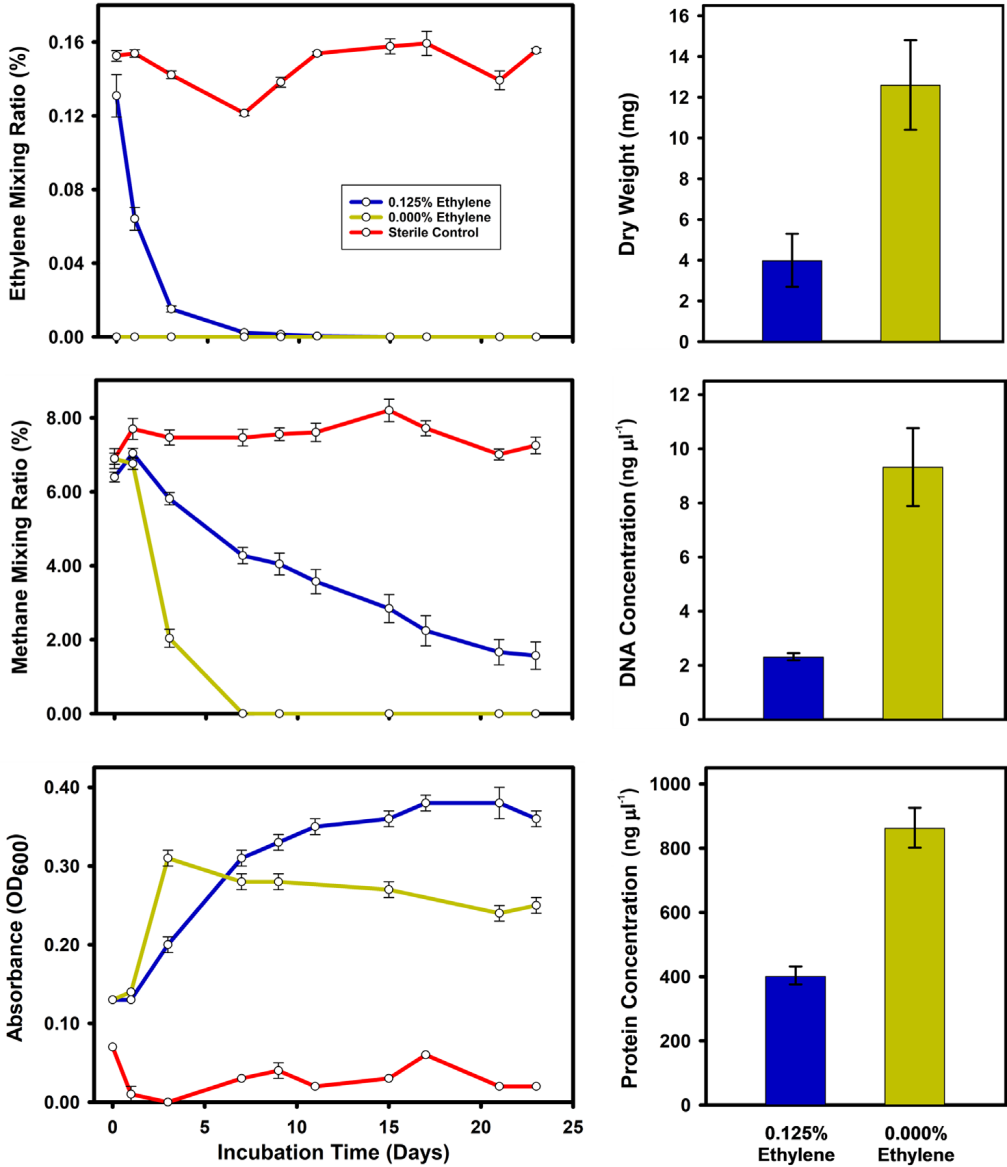
Effect of ethylene addition on *M. crimeensis* growth. Cells were grown with methane to an initial OD_600_ of 0.15 (day 0), and then ethylene was introduced to some cultures. Left panels show time courses of ethylene, methane and OD_600_. Right panels show dry weight, DNA concentration and protein concentration measurements on day 23. Data are means of six biological replicates, except the sterile control (*n*=3) and the protein concentration for the ethylene-treated cultures (*n*=3). Error bars show ±1 sem.

In cultures receiving both CH_4_ and a single dose of C_2_H_4_, CH_4_ oxidation activity and increases in OD_600_ continued after the C_2_H_4_ was consumed. Surprisingly, a higher final OD_600_ was seen in cultures receiving the C_2_H_4_ dose compared with the methane-only control cultures (Fig. 4). Although this hinted at a net energetic benefit from C_2_H_4_ oxidation (i.e. despite the slower growth, a higher final OD_600_ was produced after all substrate was consumed), we instead concluded that this was an artefact of altered cell architecture and light absorbance in C_2_H_4_-exposed cells since yields of cell dry weight, protein and DNA were all lower in the cultures exposed to C_2_H_4_ (Fig. 4). Because some CO_2_ production continued even in ethylene-inhibited cultures, we could not calculate an accurate mass balance of ethylene consumed to CO_2_ produced.

### Transcriptional and proteomics responses to ethylene

**Fig. 5.**
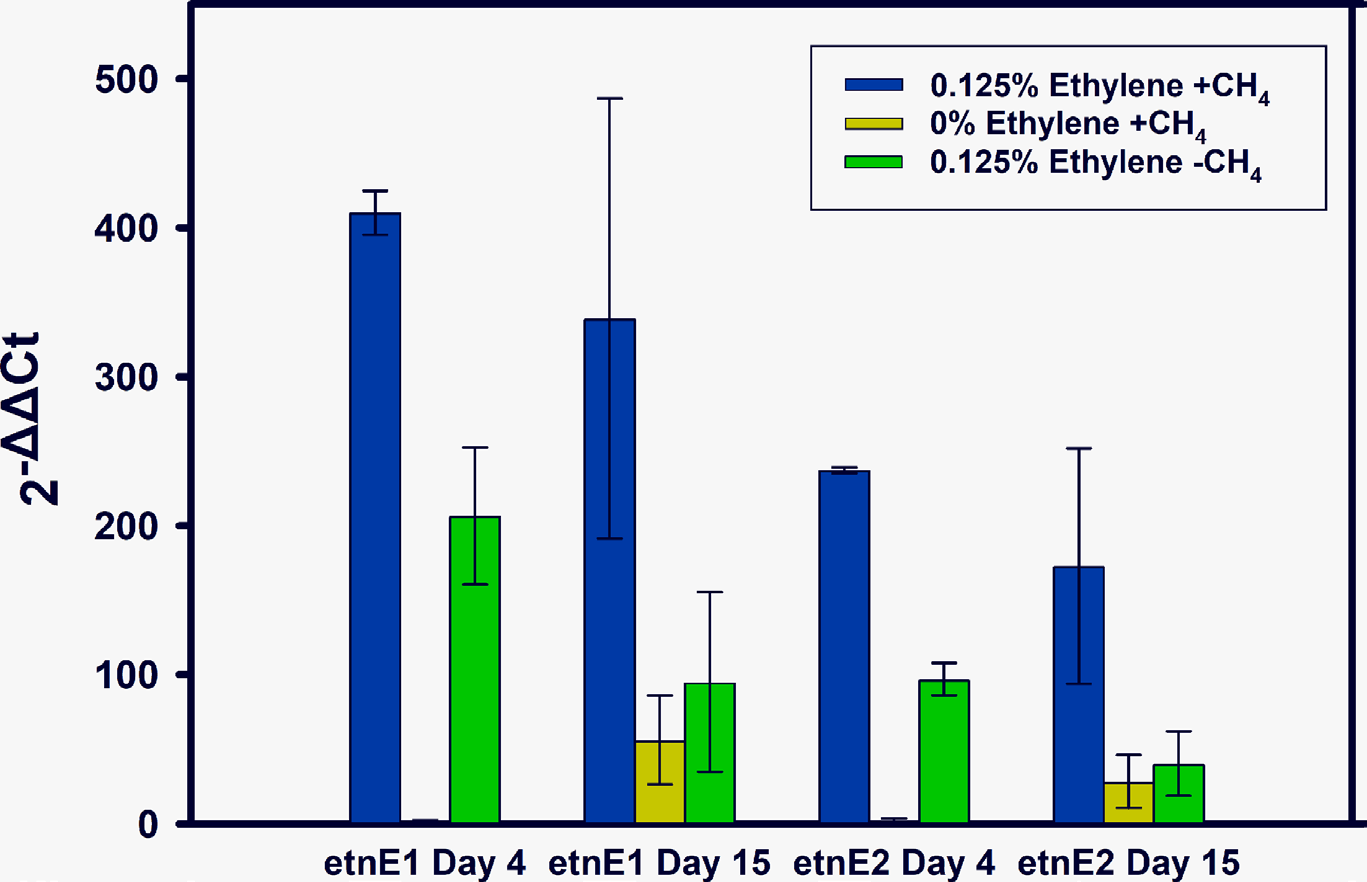
Differential expression of the *etnE* gene over time in *M. crimeensis* based on two different RT-qPCR assays (etnE1 and etnE2), in cultures exposed to 0.125% (v/v) ethylene and/or 8–10 % v/v methane. The reference *etnE* expression level is a no-ethylene treatment on day 0. Gyrase was used as a reference gene. Data are means of two biological replicates ±1 sem.

RT-qPCR of the *etnE* gene was performed on RNA extracted from three treatments: (1) CH_4_ alone, (2) C_2_H_4_ alone and (3) CH_4_+C_2_H_4_ together (Fig. 5). Cultures with C_2_H_4_ added showed higher *etnE* gene expression compared with controls without C_2_H_4_, with estimated increases up to 220-fold depending on the assay and sampling date. This C_2_H_4_ effect was highly significant with both RT-qPCR assays after 4 days (*P*<0.001) but no longer significant after 15 days. Cultures exposed to C_2_H_4_ for 15 days had ceased to grow (Fig. 3), and most cells were likely dead or dormant.

**Fig. 6.**
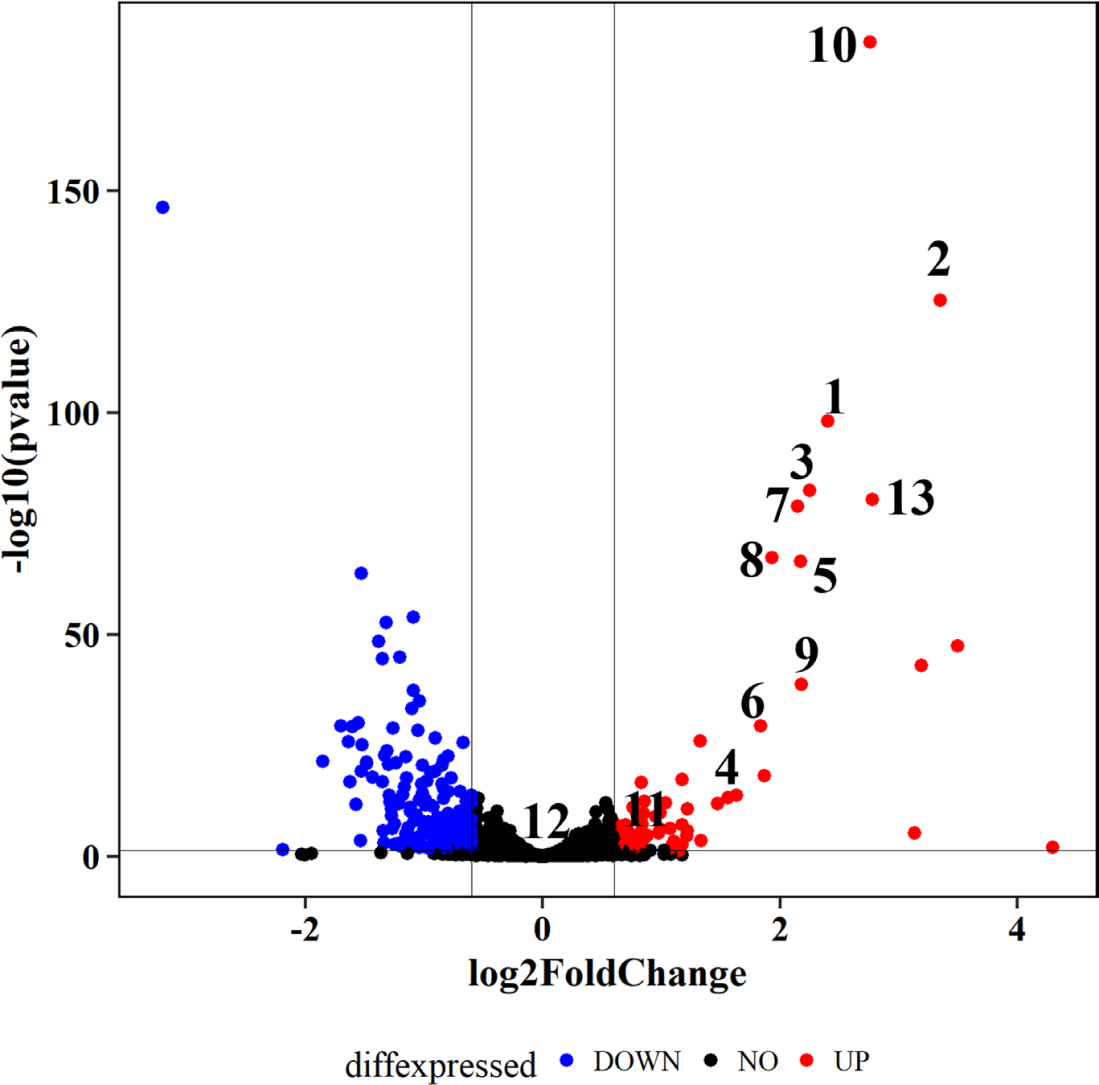
Volcano plot comparing differential protein abundance after 10 day growth of *M. crimeensis* cultures under an aerobic headspace supplemented with 8–10% CH_4_ and 0.125% C_2_H_4_ versus cultures supplemented only with 8–10% CH_4_. Putative epoxyethane metabolism/CoM biosynthesis proteins are numbered from 1 to 13 as in Fig. 1: 1, epoxyalkane:coenzyme M transferase; 2, NAD(P)-dependent hydroxypropyl CoM dehydrogenase; 3, glutaconate CoA-transferase subunit A; 4, glutaconate CoA-transferase subunit B; 5, phosphosulfolactate synthase, CoM biosynthesis protein A; 6, argininosuccinate lyase; 7, 3-carboxy-cis,cis-muconate cycloisomerase; 8, d-cysteine desulfhydrase; 9, acetyltransferase; 10, sulfoacetaldehyde dehydrogenase; 11, NADPH-dependent 2,4-dienoyl-CoA reductase, sulphur reductase; 12, MEDS MEthanogen/methylotroph, DcmR Sensory domain and 13, pyruvate/2-oxoglutarate dehydrogenase complex. All of these proteins increased in the C_2_H_4_-exposed cultures except 11–12.

The differential abundance of proteins in cultures growing on methane alone versus cultures growing on methane and then exposed to 0.125% ethylene for 10 days was assessed by LC–MS. Eleven of the 13 proteins encoded by the putative epoxyethane oxidation/CoM-biosynthesis gene cluster were shown to increase in abundance after ethylene exposure, and in fact, these 11 proteins were among the most differentially abundant proteins in the entire proteome in terms of both relative increase and significance between treatments (Fig. 6, Table S3). This gene cluster was, therefore, highly upregulated and its protein products were highly conspicuous in ethylene-challenged cells. Proteins that decreased after ethylene-challenge included many ribosomal proteins (Table S3), which is consistent with growth inhibition caused by ethylene (Fig. 3).

### Predicted etnE and related genes in other methanotrophs

Very frequently, the top 10 blastP hits of the putative epoxyethane degradation/CoM synthesis enzymes in *M. crimeensis* included other methanotrophs (Table S2). To assess the frequency with which *etnE* and related genes occur in genomes of aerobic methanotrophs, a set of 150 genomes from cultured methanotrophs of the proteobacterial classes *Alphaproteobacteria* and *Gammaproteobacteria* and phyla *Verrucomicrobiota*, *Actinomycetota* and *Ca*. Methylomirabilota (Table S4) was assembled using JGI-IMG [43] and NCBI and queried with the EtnE of *M. crimeensis* using blastP. Among these methanotroph genomes, 9.3%(14/150) possessed EtnE homologues at >65% derived amino acid identity (Table S5). Most of these methanotrophs were isolated from wetland sediments or rice paddies (Table S5). Inspection of neighbouring gene regions for each *etnE* hit revealed gene clusters similar to the one found in *M. crimeensis*. A representative selection of these gene clusters is shown in Fig. 2. There was a large identity gap between the hits to EtnE at >65% and many lower homology hits (<35%) in diverse methanotroph genomes. These low homology hits are probably methionine synthase and did not show any similarities in adjacent gene arrangements.

### Evidence for lateral gene transfer

**Fig. 7.**
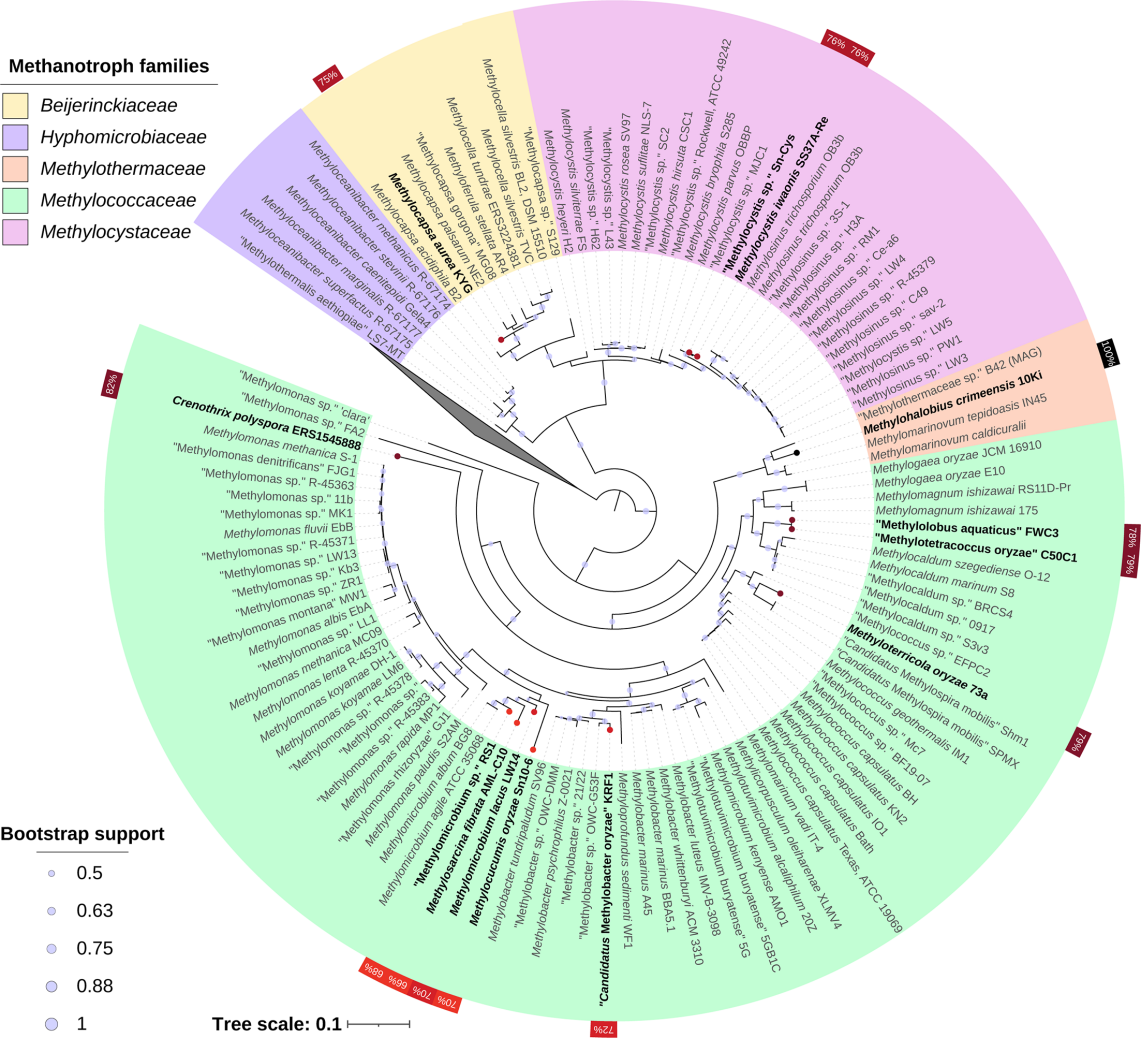
Phylogenetic reconstruction based on 16S rRNA genes of the methanotrophic bacteria listed in Table S4. The tree was inferred by FastTree v2.1.11 using the generalized time-reversible model of nucleotide substitution and 1000 bootstrap reconstructions. The non-proteobacterial sequences were used as an outgroup and are displayed as a collapsed node. The taxa are colour-coded at the family level. Genomes containing *etnE* gene homologues are highlighted in bold and by red dots at the tips. The red tabs on the outside of the circle display derived amino acid identity to the *etnE* gene in *M. crimeensis* 10Ki.

Within the phylum *Pseudomonadota* (*Proteobacteria*), aerobic methanotrophs occur in 2 classes (*Alphaproteobacteria* and *Gammaproteobacteria*), 5 families (*Methylocystaceae*, *Beijerinckiaceae*, *Hyphomicrobiaceae*, *Methylococcaceae* and *Methylothermaceae*), 25 genera and 72 species [61]. These can all generally be delineated as monophyletic groups (e.g. [62,63]). The *etnE* gene was found in only 14 of 150 methanotroph genomes analysed, but this included bacteria from both proteobacterial classes, 4 of 5 families and 9 of 25 genera (Tables S4–S5). With the caveat that available genomes do not cover all methanotrophic taxa, we can nevertheless conclude that *etnE* is broadly distributed across distant phylogenetic lineages of methanotrophs but also rare (Fig. 7). Furthermore, within each genus, only a low fraction of genomes contain *etnE*. For example, of 16 *Methylocystis* genomes examined, only 2 contained *etnE*. Of five *Methylocapsa* genomes, only 1 contained *etnE* (Tables S4–S5). Besides *M. crimeensis* strain 10Ki, no other genomes of the taxonomic family *Methylothermaceae* contained these genes. Given this scattered, mosaic phylogenetic occurrence (Fig. 7), it is obviously more parsimonious to conclude that genes encoding epoxyethane catabolism were transferred laterally several times into methanotrophic bacteria rather than lost in the vast majority of lineages.

Each derived protein in the ethylene catabolism/CoM synthesis-encoding gene cluster was also examined via blastP against the NCBI nr database (Table S2). The top hit for most genes was ‘*Betaproteobacteria* bacterium HGW-Betaproteobacteria-11 (groundwater metagenome)’ [64]. However, because this is a MAG bin and the genes are likely subject to LGT, this taxonomic match should be considered uncertain. A consistent taxonomic pattern of blast hits was not evident, top hits were to a mixture of *Betaproteobacteria* (*Burkholderiales* or *Rhodocyclales*), *Gammaproteobacteria*, *Alphaproteobacteria*, *Chloroflexota*, *Myxococcota* and *Actinomycetota*, indicating that the different genes may have different origins. The lack of close homologues from a pure culture prevents us from determining the likely source(s) of the LGT event(s) into *M. crimeensis*.

**Fig. 8.**
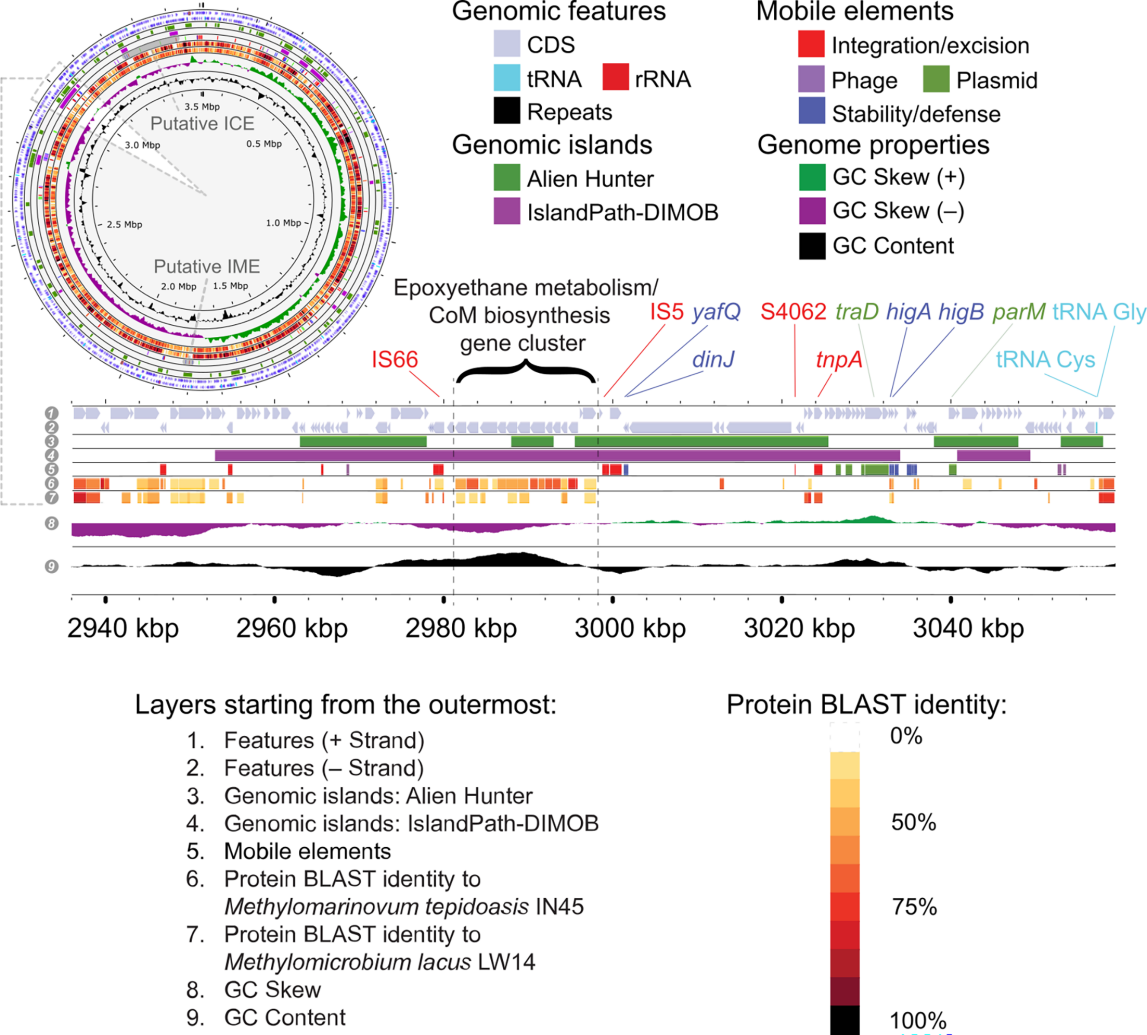
Genes encoding epoxyethane metabolism and CoM biosynthesis in *M. crimeensis* 10Ki are located in a genomic region showing strong compositional evidence of lateral gene transfer. Layers in the circular genome (upper left) and in an enlargement of the region containing the gene cluster of interest (bottom) indicate (1–2) features of the *M. crimeensis* genome; (3–4) genomic islands predicted by two methods; (5) mobile elements detected with mobileOG-db v1.1.3 [47] or annotated by JGI-IMG; (6–7) protein homology of *M. crimeensis* coding regions to *Methylomicrobium lacus* LW14 (close taxonomic neighbour containing the genes of interest) and *Methylomarinovum tepidoasis* IN14 (close taxonomic neighbour lacking the genes of interest); (8–10) GC skew and GC content of *M. crimeensis*. Genes encoding epoxyethane metabolism are labelled at the top, along with some mobility genes and insertion sequences (a full list is given in Table S6). The five contigs of the *M. crimeensis* genome were concatenated, and the genomic island is located on the second largest contig (737 379 bp). A predicted integrative mobilizable element (IME) and integrative and conjugative element (ICE) are also indicated on the full genome diagram. The diagrams were produced using Proksee [84].

Multiple non-phylogenetic methods were also applied to predict LGT in *M. crimeensis*. The entire gene cluster encoding epoxyethane metabolism was part of a large (c. 100  kb) genomic island predicted by IslandPath DIMOB, which assesses dinucleotide frequencies and the presence of mobility genes. This island was largely confirmed by AlienHunter, which assesses genomic variation in k-mer frequencies between 2-mers and 8-mers (Fig. 8). The predicted genomic island is characterized by an irregularity in the GC skew and contains a large number of mobility elements including transposases, conjugation factors, insertion sequences and virulence factors (Fig. 8, Table S6). IS66 and IS5 were found immediately flanking the *entE*-containing gene cluster. A large cluster of genes in the genomic island, beginning 27 kb upstream of *etnE*, show high homologies to genes typical of ICEs, specifically several ICE proteins of the PFGI-1 ICE class as in *Pseudomonas,* a toxin-antitoxin system that could be involved in ICE maintenance, and regulatory elements for type IV pili. ICEBerg [48] failed to identify this genomic island as an ICE, although it did predict a large ICE nearby on the same contig (Fig. 8).

When comparing *M. crimeensis* 10Ki to the reference methanotroph genomes *Methylomicrobium lacus* LW14 (close taxonomic neighbour containing *etnE*) and *Methylomarinovum tepidoasis* IN14 (close taxonomic neighbour lacking *etnE*), there is a high percentage of homologous genes across most of the genome, reflecting the common core genomic repertoires of obligate aerobic methanotrophs (Fig. 8). However, the genomic island region highlighted in Fig. 8 contains few homologues to either reference genome, even the *Methylomicrobium lacus* LW14 strain that also contains *etnE*. Because *M. crimeensis* displayed such strong evidence for LGT of the epoxyethane-degradation gene cluster, AlienHunter and IslandPath-DIMOB were also applied to all methanotroph genomes containing *etnE*. However, only the gene clusters in *M. crimeensis* and * M. aurea* were detected by the programs as foreign (Table S5). Although we predict based on phylogenetic distribution that epoxyethane metabolism has likely been acquired via LGT in all methanotrophs that possess this function (Fig. 7), only two strains show strong compositional genomic evidence for this LGT. These may be the most recent transfer events.

## Discussion

To date, genes encoding epoxyethane metabolism have been studied only in organoheterotrophic bacteria that use alkenes as carbon and energy sources for growth, including species of *Xanthobacter*, *Haliea*, *Mycobacterium*, *Nocardioides*, *Rhodococcus *and *Pseudomonas* [12,26, 56, 65,69]. Here, we document the upregulation of genes encoding epoxyethane oxidation after exposure of the obligate methanotroph *Methylohalobius crimeensis* to ethylene. In this bacterium, ethylene does not seem to provide any energetic benefit but is instead toxic. We further suggest that this phenomenon is not unique to *M. crimeensis* but is present in some other methanotrophs that inhabit suboxic soils and sediments where natural ethylene production could impact their growth on methane.

The biotoxicity of ethylene in all organisms is due to the high reactivity of its primary oxidation product epoxyethane (ethylene oxide). This epoxide is a strong electrophile and readily undergoes ring opening reactions with cellular components, thus damaging the cell [30, 17] and acting as a mutagen [70]. For example, exposure to only 10 ppmv ethylene has been shown to damage plant tissues [29]. The highly reactive epoxyethane is expected to form whenever methane oxidation takes place in the presence of ethylene, as a consequence of both the pMMO and sMMO enzymes’ capacity to co-oxidize short-chain alkenes [14,16]. Therefore, the presence of ethylene poses an existential threat to all methanotrophs.

In our study, ethylene addition to *M. crimeensis* cultures always had a negative effect on cell activity and growth, but the degree of inhibition was dependent on the amount and duration of ethylene exposure. We propose that the capacity for epoxyethane metabolism is the likely reason for this methanotroph’s ability to grow in the presence of some ethylene. The pathway of ethylene oxidation in *M. crimeensis* was predicted based on comparative genomic analysis with bacteria that grow on alkenes as carbon and energy sources, *Mycobacterium rhodesiae* strain JS60 [26, 28] and *Xanthobacter autotrophicus* Py2 [56,57]. It should be noted that even in these well-studied bacteria, the evidence supporting alkene metabolism and CoM biosynthetic pathways is incomplete, with only a few steps confirmed experimentally. However, there was a close correspondence between the gene repertoires encoding epoxyethane metabolism in *M. crimeensis* and the two reference strains (e.g. Fig. 2), with high sequence identities of the corresponding homologues (Table S2). These genes encode a series of metabolic steps (epoxide conjugation to CoM mediated by the EtnE enzyme, followed by conversion of epoxide to acetyl-CoA), along with biosynthesis of CoM, and a small molecule-sensing regulatory protein (Fig. 1). The major difference between the corresponding gene repertoires is the presence of genes encoding alkene monooxygenase in the reference strains. These genes are missing in the obligate methanotroph *M. crimeensis*, where the only realistic mechanism for ethylene oxidation is unintended co-oxidation via pMMO.

We, therefore, hypothesize that when ambient ethylene levels are high, the EtnE-mediated pathway relieves the toxicity of the incidental epoxides that are inevitably produced by pMMO. Several lines of experimental evidence support this hypothesis. Firstly, ethylene consumption (at 0.125% v/v) was observed by gas chromatography in *M. crimeensis* cultures, during which growth and methane oxidation were slowed but not completely inhibited (Figs3  4). In cultures where ethylene was added a second time, the decrease in OD_600_ and cessation of methane oxidation suggests that the ethylene tolerance of the cultures had been exceeded (Fig. 3). Secondly, RT-qPCR analysis of *etnE* showed that this gene was strongly transcriptionally upregulated in the presence of ethylene (Fig. 5). Thirdly, proteomics data verified that EtnE and 10 of the other proteins in the same gene cluster became much more abundant in cells after exposure to ethylene. It is notable that the *etnE*-containing gene cluster detected in *M. crimeensis* and other methanotrophs encodes all the enzymes predicted to be needed for CoM biosynthesis (Figs1  2), highlighting the special role of CoM in small hydrocarbon metabolism [56]. CoM biosynthesis genes have been found to date only in methanogenic *Archaea* and aerobic alkene-utilizing bacteria, thus, their presence in *M. crimeensis* is strong circumstantial evidence for its capability to metabolize alkene-derived epoxides [57].

Ethylene levels of 1–15 ppmv have been measured in soil gas pores [24,25]. Ethylene is usually highest in waterlogged, O_2_-depleted soils and sediments due to increased production rates and reduced diffusion rates [24,71, 72]. Anaerobic soil microcosms can yield equilibrium headspace concentrations as high as 20 ppmv [72,73]. The high production of ethylene in anoxic conditions has been attributed to the action of an anaerobic bacterial sulphur assimilation pathway that uses nitrogenase-like enzymes to break C-S bonds in volatile organic sulphur compounds, releasing ethylene as a waste product [20]. Suboxic and anoxic soils and sediments are, therefore, sites of elevated ethylene production. Such soils are also methanogenic, and their aerobic interfaces are habitats for methanotrophic bacteria. Based on our survey of methanotroph genomes, methanotrophs possessing putative epoxyethane metabolic genes (including *M. crimeensis*) usually inhabit waterlogged sediments. This suggests that the simultaneous production of elevated amounts of ethylene and methane presents a challenge to methanotrophs and that an epoxyethane detoxification pathway can confer adaptive value.

Ethylene levels similar to those detected in natural soils (3–50 ppmv) were shown to strongly inhibit methane oxidation in several forest soils [31,32]. Interestingly, the genome of the sole cultured representative of a methanotroph species frequently detected in high abundance in most acidic forest soils, *Methylocapsa gorgona* MG08 [74], does not have a close homologue of *etnE* or other epoxyethane metabolic genes and should, therefore, be prone to ethylene inhibition. In contrast, *M. crimeensis* should be well equipped to deal with natural levels of ethylene. Cultures were able to survive and oxidize ethylene at mixing ratios of 0.125% v/v or below (1250 ppmv), which is two orders of magnitude higher than levels typically measured in soils or sediments. However, organoheterotrophic bacteria that utilize alkenes for growth can tolerate much higher concentrations. For example, *Haliea* strains ETY-M and ETY-NAG from seawater were isolated using atmospheres containing 50% v/v ethylene [12], while *M. rhodesiae* JS60 is grown routinely under 10% v/v ethylene [26]. The lower tolerance of *M. crimeensis* compared with these dedicated alkene consumers could be related to energetic considerations, i.e. at the ethylene concentrations used in our experiments, the bacterium appears to derive no energetic benefit from ethylene. The competitive nature of methane and ethylene on the pMMO active site implies that methane must be present in much higher levels than ethylene to optimize the process of energy generation over toxin production.

*M. crimeensis* fixes one-carbon substrates using the ribulose monophosphate cycle but cannot grow on two-carbon substrates alone [34]. This would normally require either the glyoxylate cycle or the ethylmalonyl-CoA pathway [75]. The absence of isocitrate lyase and malate synthase genes confirms that the glyoxylate shunt is absent in *M. crimeensis*, while the absence of genes for ethylmalonyl-CoA epimerase, (2*R*)-ethylmalonyl-CoA mutase and (2*S*)-methylsuccinyl-CoA dehydrogenase indicates the absence of the ethylmalonyl-CoA pathway [35]. The organism does encode a putative pyruvate:ferredoxin oxidoreductase (H035DRAFT_0892–0893, 2641, 2805). The use of a ferredoxin-requiring assimilation pathway in an aerobic organism is generally considered unlikely, although recent evidence suggests that this enzyme is O_2_ tolerant and can sometimes operate under aerobic conditions [76]. Nevertheless, even if the organism has no means for net growth from two-carbon substrates alone, NAD(P)H and acetyl-CoA produced via ethylene oxidation could still benefit the cells. Acetyl-CoA could simply be oxidized to produce energy (and/or more NAD(P)H reducing equivalents) to support growth on methane-derived carbon. Alternatively, acetyl-CoA could be used directly in the biosynthesis of fatty acids or certain amino acids. The genome encodes a putative acetyl-CoA (de)carboxylase (H035DRAFT_0892–0893, 2641, 2805) to produce the fatty acid precursor malonyl-CoA and a complete downstream pathway for fatty acid metabolism. If the end product of ethylene oxidation is indeed acetyl-CoA, this will mix with the cellular pool of acetyl-CoA, and thereby some of the ethylene-derived carbon will almost certainly become assimilated into biomolecules. In our experiment, the net growth effect of ethylene addition was negative, presumably because the toxicity of epoxyethane represented an energy sink that outweighed any of the potential catabolic or anabolic benefits of the produced NAD(P)H and acetyl-CoA. However, smaller amounts of ethylene, similar to those present in natural flooded soils, could incur correspondingly lower epoxide toxicity and shift the balance between growth inhibition and growth stimulation to a net positive, a possibility we are currently researching. Although using a lower ethylene concentration is environmentally more relevant, a higher ethylene concentration was chosen in these initial studies for experimental convenience, as it increased our ability to measure ethylene consumption, gene expression, proteomics and growth effects. We note in our incubation time courses that headspace ethylene passed through the natural range of 3–20 ppm on the way to zero (Fig. 4). Therefore, cultures are capable of consuming lower, natural mixing ratios of ethylene.

The presence of epoxyethane metabolism-encoding genes in methanotrophs poses questions about their mobility. The phylogenetic distribution of the genes, in which they occur rarely but are scattered across several distinct monophyletic taxa, suggests multiple LGT events in methanotrophs. In *M. crimeensis*, these genes appear to be located on a large genomic island that shows abundant compositional evidence of LGT. The mechanism of transfer could not be demonstrated for certain, although some evidence indicated the involvement of an ICE, which often incorporates cargo genes unrelated to the ICE life cycle [77]. This is similar to the situation in species of the phyla *Actinomycetota* and *Pseudomonadota* (or *Proteobacteria*) that grow on ethylene and propylene, where the alkene and epoxide catabolic genes are found on large, highly mobile plasmids [78,80].

We predict that the expression of the genes encoding epoxyethane oxidation in *M. crimeensis* is under the control of a small molecule sensing protein containing a MEDS domain along with a helix-turn-helix DNA-binding domain. The MEDS domain is typical of regulatory proteins like DcmR that bind to simple hydrocarbon derivatives and then transduce regulatory signals [58,59]. Interestingly, this regulatory gene is not present in any of the gene clusters detected in other methanotrophs. It is also somewhat distinct from the traditional two-component regulatory system (EtnR1, EtnR2) used by mycobacteria to regulate growth on ethylene, which specifically detects the epoxide and acts at a cognate promoter sequence [60]. There is a high homology of the MEDS domain of the regulatory proteins in *M. crimeensis* and JS60, but the DNA binding mechanism is likely distinct. Notably, the MEDS domain is also hypothesized to function as a negative regulator of the sigma factor SigB in actinobacteria, which is involved in the stress response regulon [58]. The *M. crimeensis* genome encodes a sigma factor considered a functional homologue of SigB in Gram-negative bacteria, sigma-38 or RpoS [81]. This is a member of the diverse sigma-70 family and a global regulator of stress response [82]. In response to stress conditions, sigma-38 (RpoS) can partially replace the sigma-70 (RpoD) subunit in binding to the bacterial RNA polymerase core enzyme. Differences in promoter recognition between RpoD and RpoS are difficult to detect but have been suggested to be related to factors such as the effects of additional transcription factors, DNA topology, the spacing between the −35 and the −10 elements and the presence of complete or partial UP element sequences [83]. However, our prediction of binding sites for a homologue of the stress response regulator CpxR in the target operon in *M. crimeensis* supports a hypothesis that this operon is likely expressed in response to oxidative stress. As a result of oxidative stress from epoxyethane, RpoD might be displaced by RpoS to initiate transcription of the operon for epoxyethane degradation. Further experimentation will be needed to determine the roles of sigma factors and the MEDS small molecule sensing protein in regulating the response to epoxyethane in *M. crimeensis*. The absence of the MEDS domain regulatory protein in the corresponding gene clusters of other methanotrophs (Fig. 2) suggests that the regulation in *M. crimeensis* may be unique.

## Conclusions

The obligate methanotroph *M. crimeensis* was shown to consume ethylene at a mixing ratio of 0.125% v/v during growth on methane but was not capable of growth on ethylene alone, nor of deriving a growth benefit from ethylene in the presence of methane, at least not at the mixing ratios we used. A gene cluster putatively encoding CoM biosynthesis and CoM-dependent epoxyethane metabolism was found, similar to gene clusters in bacteria that grow on alkenes as energy and carbon sources. The key gene *etnE* was highly upregulated in *M. crimeensis* after ethylene exposure, and 11 of the 13 proteins encoded by the predicted *etnE*-containing gene cluster greatly increased in abundance, strongly implicating their role in ethylene response. Similar gene clusters were found in about 10% of sequenced genomes of aerobic methanotrophs, all from wetlands or other suboxic habitats. We propose that the CoM-mediated epoxide metabolism provides an important detoxification mechanism for methanotrophs inhabiting niches rich in both methane and ethylene, where co-oxidation of ethylene by the promiscuous pMMO enzyme will produce potentially harmful epoxides.

Biomass yields of *M. crimeensis* were always negatively affected by ethylene exposure, at least at the levels tested here. However, the net gain of acetyl-CoA via the epoxide oxidation pathway implies that smaller doses of ethylene might contribute to cell energetic balance or that other methanotrophs with more versatile catabolic repertoires (like some *Methylocystis* and *Methylocapsa* species) might be capable of using ethylene as a supplementary energy source while growing on methane. Further work is required to compare different methanotrophs and to confirm issues such as the primary role of these enzymes in detoxification, the biochemical steps in CoM biosynthesis and epoxide metabolism and the exact fate of carbon derived from ethylene. An obvious direction is to create knockout mutations. Unfortunately, tools for genetic manipulation of *M. crimeensis* have not been developed, making this a difficult proposition. However, the presence of multiple closely related cultivated strains of *Methylocystis*, some with and some without the genomic capacity for epoxide oxidation, should allow simple comparisons to be made about ethylene responses within this genus.

## supplementary material

10.1099/mgen.0.001306 Uncited Fig. S1.

10.1099/mgen.0.001306 Tables S1 to S6.

## Abbreviations

CoM: coenzyme-M
CuMMO: copper membrane monooxygenase
HCD: higher energy collisional dissociation
ICEs: integrative and conjugative elements
JGI-IMG: Joint Genome Institute Integrated Microbial Genomes
LC–MS: liquid chromatography–mass spectrometry
LGT: lateral gene transfer
MEDS: methanogen/methylotroph, DcmR sensory
MMO: methane monooxygenase
NCBI: National Center for Biotechnology Information
pMMO: particulate membrane-bound form
RT-qPCR: reverse transcription-quantitative PCR
SDIMO: soluble di-iron-containing multicomponent monooxygenase family
sMMO: soluble cytoplasmic form
TMT: tandem mass tag
