# Supplementary material for: Ethylene and epoxyethane metabolism in methanotrophic bacteria: comparative genomics and physiological studies using Methylohalobius crimeensis
Source: Microb Genom. 2024 Oct 25;10(10):001306. doi: 10.1099/mgen.0.001306 (PMC11507031; doi:10.1099/mgen.0.001306)

Supplementary Figure S1. Inhibitory effect of ethylene on *M. crimeensis* cultures growing on methane. Ethylene was added to the headspaces on day 6 at 0.5-5% (v/v). A) OD_{600} of cultures B) Methane concentration in culture headspaces. Data are means of 4 biological replicates ± 1 SEM. Ethylene + low methane cultures had their atmospheres partially flushed prior to ethylene addition to reduce their methane mixing ratios.

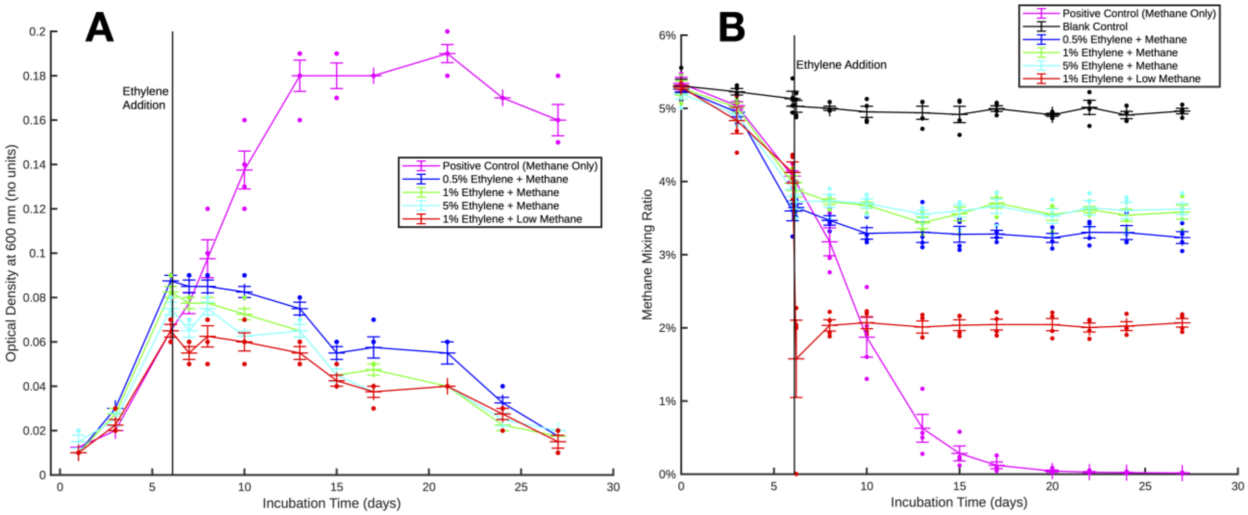

Supplement: Uncited Fig. S1. [file mgen-10-01306-s001.pdf]
